# Peer Review of “Identifying Safeguards Disabled by Epstein-Barr Virus Infections in Genomes From Patients With Breast Cancer: Chromosomal Bioinformatics Analysis”

**DOI:** 10.2196/70039

**Published:** 2025-01-29

**Authors:** Anonymous

**Keywords:** breast, cancer, oncology, ovarian, virus, viral, Epstein-Barr, herpes, bioinformatics, chromosome, gene, genetic, genetics, chromosomal, DNA, genomic, BRCA, metastasis, biology

*This is the peer-review report for “Identifying Safeguards Disabled by Epstein-Barr Virus Infections in Genomes From Patients With Breast Cancer: Chromosomal Bioinformatics Analysis.”*

## Round 1 Review

### Review Report With Major Revisions for the Paper

Title: “Herpesvirus infections eliminate safeguards against breast cancer and its metastasis: comparable to hereditary breast cancers”

### Summary

The paper [1] hypothesizes that Epstein-Barr virus (EBV) infections promote breast cancer by disabling cancer safeguards. It is a bioinformatics analysis of public information from about 2100 breast cancers. The study finds that breast and ovarian cancer breakpoints cluster around EBV-associated cancer breakpoints, suggesting a significant role of EBV in promoting these cancers. The paper also identifies similarities in the molecular and cellular disruptions caused by EBV with those found in hereditary breast cancers.

### Major Revisions Needed

#### Clarification of Hypotheses and Objectives

The hypothesis, while intriguing, needs clearer articulation. Specifically, the connection between EBV and breast cancer needs more explicit theoretical underpinning. Clarify the objectives and expected outcomes of the study at the outset.

#### Methodological Rigor and Data Sources

While the bioinformatics approach is robust, it would benefit from a more detailed description of the methods and algorithms used. Additionally, the selection criteria for the breast cancer data should be justified more thoroughly to avoid selection bias.

#### Statistical Analysis

The statistical methods used need more comprehensive detailing. For complex analyses, ensure the statistical assumptions and any transformations of data are clearly explained. Include more information on the statistical tests used for hypothesis testing and the justification for their use.

#### Comparative Analysis

The comparison between hereditary breast cancers and those potentially caused by EBV is insightful. However, a more detailed comparative analysis would strengthen the argument. This could include molecular or genetic profiling comparisons.

#### Discussion on Contradictory or Supporting Evidence

The discussion section should address not only the supporting evidence but also any contradictory findings in the literature. This balance is crucial for a nuanced understanding of the subject.

#### Implications and Future Research Directions

The implications of these findings are profound but need clearer articulation. Discuss the potential impact on breast cancer treatment and prevention strategies. Also, outline future research directions, particularly in clinical or experimental studies, to confirm these bioinformatics findings.

#### References

Please add more background information about breast cancer (please cite: 1. Cao Y, Efetov S, He M, et al. Updated clinical perspectives and challenges of chimeric antigen receptor-T cell therapy in colorectal cancer and invasive breast cancer. Arch Immunol Ther Exp (Warsz). Aug 11, 2023;71(1):19. [doi: 10.1007/s00005-023-00684-x] [Medline: 37566162]; and 2. Liu Y, Lu S, Sun Y, et al. Deciphering the role of QPCTL in glioma progression and cancer immunotherapy. Front Immunol. Mar 29, 2023;14:1166377. [doi: 10.3389/fimmu.2023.1166377] [Medline: 37063864]).

### Concluding Remarks

The paper presents a novel and potentially significant hypothesis linking EBV to breast cancer. However, it requires major revisions to enhance its methodological rigor, clarity, and comprehensiveness. Addressing these concerns will significantly strengthen the manuscript’s impact and contribution to the field.

## Abbreviations

EBV: Epstein-Barr virus
